# Percutaneous ultrasound-guided versus open cut-down access to femoral vessels for the placement of a REBOA catheter

**DOI:** 10.1038/s41598-024-59778-x

**Published:** 2024-04-20

**Authors:** Peter Grechenig, Barbara Hallmann, Nicolas Rene Eibinger, Amir Koutp, Paul Zajic, Gerald Höfler, Paul Puchwein

**Affiliations:** 1 Department of Orthopedics and Trauma Surgery, Medical University of Graz, Graz, Austria; 2 Department of Anaesthesiology and Intensive Care Medicine, Medical University Graz, Auenbruggerplatz 5, 8036 Graz, Austria; 3 Diagnostic and Research Institute of Pathology, Medical University Graz, Graz, Austria

**Keywords:** Translational research, Trauma

## Abstract

Resuscitative Endovascular Balloon Occlusion of the Aorta (REBOA) may be useful in treating exsanguinating trauma patients. This study seeks to compare rates of success, complications and time required for vascular access between ultrasound-guidance and surgical cut-down for femoral sheath insertion as a prospective observational case control study. Participating clinicians from either trauma surgery or anesthesiology were allocated to surgical cut-down or percutaneous ultrasound-guided puncture on a 1:1 ratio. Time spans to vessel identification, successful puncture, and balloon inflation were recorded. 80 study participants were recruited and allocated to 40 open cut-down approaches and 40 percutaneous ultrasound-guided approaches. REBOA catheter placement was successful in 18/40 cases (45%) using a percutaneous ultrasound guided technique and 33/40 times (83%) using the open cut-down approach (*p* < 0.001). Median times [in seconds] compared between percutaneous ultrasound-guided puncture and surgical cut-down were 36 (18–73) versus 117(56–213) for vessel visualization (*p* < 0.001), 136 (97–175) versus 183 (156–219) for vessel puncture (*p* < 0.001), and 375 (240–600) versus 288 (244–379) for balloon inflation (*p* = 0.08) overall. Access to femoral vessels for REBOA catheter placement is safer when performed by cut-down and direct visualization but can be performed faster by an ultrasound-guided technique when vessels can be identified clearly and rapidly.

## Introduction

Resuscitative Endovascular Balloon Occlusion of the Aorta (REBOA) for exsanguinating patients is a comparably old intervention in medicine^1^. Advances in technology and consensus on the need for swift bleeding control in trauma resuscitative care within the last decade have renewed clinical and scientific interest in the technique^2,3^. REBOA has been proven a useful adjunct in the resuscitative care of trauma patients although there is distinct lack of evidence on the subject^4,5^.

Complications after the use of REBOA have led to the conclusion that its use could even be detrimental^6^. Several case-series report at least half of complications to be associated with femoral access for catheter placement^7^. Issues encompass complications at the site of access itself, ischemia due to hampered limb perfusion, and inability to achieve access in a timely fashion^8^. The UK-REBOA Trial has been published recently and concerns about a possible increased mortality have been raised, with the main concern that the procedure could delay definitive treatment^9^. To this day there is no clear evidence to guide clinicians on which technique for vascular access should be preferred, to overcome this issue^10^.

Possible techniques to achieve access to the femoral artery encompass landmark- and palpation-based vessel puncture, ultrasound-guided vessel puncture, and surgical cut-down towards the femoral vessels. These approaches come with different prerequisites for providers’ skillsets and training and may be associated with different rates of success and complications. Widespread availability of ultrasound and its superior safety profile over landmark-based blind palpation-only puncture, renders it the favorable method nowadays for vascular access in critically ill in general^11,12^ and REBOA especially^13^.

### Aim of this study

In this study, we primarily seek to compare the rates of success and complications between ultrasound-guided femoral sheath insertion and surgical cut-down for sheath insertion in a close to (real) life model. Secondly, we aimed to elucidate, whether there are differences between approaches in the time needed to achieve vascular access.

## Methods

### Study design

We designed a prospective observational case control study using a cadaveric training model using the STROBE guideline^14^. The CACTUS guideline was used to ensure proper reporting of cadaver characteristics^15^. Participants were allocated to an open surgical cut-down approach or a percutaneous ultrasound-guided approach to place the REBOA catheter on a 1:1 ratio.

### Ethical approval

Approval from ethics committee at the Medical University Graz was sought and granted (34-247 ex 21/22) in accordance with the principles of the Declaration of Helsinki and the ICH-GCP Guidelines.

### Participants

Study participants were physicians recruited from the University Medical Centre Graz (Level-1-Trauma Centre). All participants were involved in the emergency management of major trauma patients either in the emergency department or in the prehospital setting. Training of participants ranged from junior residents to senior attendings. Participants were recruited from medical specialties tasked with treatment of exsanguinating trauma patients in our trauma system, i.e., Orthopedics and Trauma Surgery and Anesthesiology and Intensive Care Medicine (Table 1).

**Table 1:** Characteristics of participants overall and compared between the percutaneous ultrasound-guided technique group and the surgical cut-down access group.

	Overall	Percutaneous ultrasound-guided technique	Surgical cut-down access
*n* of participants	80	40	40
Medical specialty (n, %)			
Anesthesiology and Intensive Care Medicine	48 (60%)	40 (100%)	8 (20%)
Orthopedics and Trauma Surgery	32 (40%)	0 (0%)	32 (80%)
Training status (n, %)			
Resident	27 (34%)	17 (43%)	10 (25%)
Specialist	26 (32%)	11 (27%)	15 (37.5%)
Attending	27 (34%)	12 (30%)	15 (37.5%)

Participant allocation to different procedure-groups was done based on specialty: surgically trained personnel were designated to the open cut-down access, non-surgically trained personnel were allocated to the percutaneous ultrasound group. This group allocation was chosen to assign participants the approach they would most likely be tasked with in our trauma resuscitation setting and in accordance with Austrian physician training standards to ensure real- life conditions. Each participant was theoretically trained beforehand with lectures and simulation training, and got another on-site lecture on the task to be performed and familiarization time with the equipment used, before starting the procedure (Table 1).

### Setting and Cadaver Specifics

For this study freshly cooled cadavers were used, storage was between − 1 to + 6 °C until half an hour before the training. The investigated procedures on the cadavers were performed within 48 h after death. The procedure site was the dissecting room at the Institute for Pathology at the Medical University Graz. 43 cadavers were used during the study period.

Median (IQR) age of the deceased was 76 years (62–80) [minimum 29, maximum 85 years] years, median BMI was 27 (26–29) [minimum 19, maximum 39] kg/m^2^ with 16 cadavers having visual and tactile arterial calcification indicating severe peripheral artery disease. (Table S1—Additional File 1) Cadavers with previous surgery to the inguinal region or devices placed in the femoral artery were not included in this study. During the procedure, only participants and investigators were present out of respect for the human body.

### Procedure

**Figure 1:**
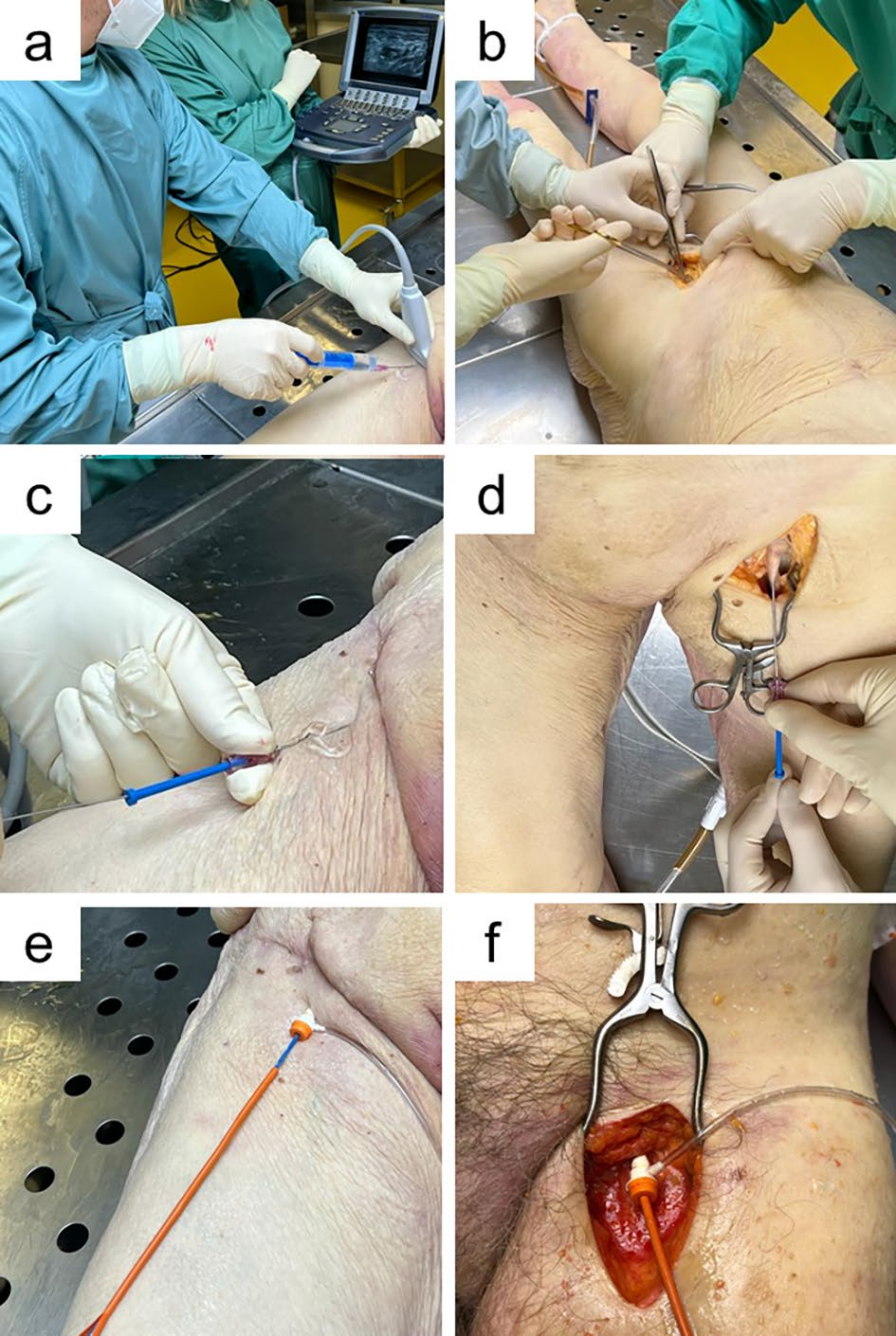
Conduction of REBOA (Resuscitative Endovascular Balloon Occlusion of the Aorta) according to study procedure and equipment use. Top row: femoral vessel visualization by (**a**) ultrasound-guidance using a SonoSite® M-Turbo Series ultrasound machine and a 15-6 MHz linear probe and (**b**) surgical cut-down using surgical forceps, scalpel, wound distractors, surgical scissors for open cut-down access; Middle row: femoral artery puncture and guidewire introduction following (**c**) ultrasound-guidance and (**d**) surgical cut-down; Bottom row: after balloon inflation following (**e**) ultrasound-guidance and (**f**) surgical cut-down.

For the cut-down approach, participants were instructed to perform a longitudinal incision beginning midway between the superior iliac spine and the pubic symphysis, extending over the medial border of the sartorius muscle, and proceeding toward the medial femoral epicondyle. After incision of the subcutaneous tissue and fascia lata, the femoral sheath was incised to expose the vessels. The following instruments were used to implement the open cut-down approach: surgical forceps, scalpel, wound distractors, surgical scissors. (Fig. 1).

For the percutaneous ultrasound-guided approach, participants were instructed to identify the femoral vessels at the level of the inguinal ligament with recommended puncture site below the inguinal ligament. For visualization and needle-guidance a 15–6 MHz linear probe and a SonoSite® M-Turbo Series ultrasound machine was used. (Fig. 1).

For both procedures, cannulation of the vessel was performed using a 7 French introducer sheath. For balloon occlusion of the aorta, ER-REBOA catheters (Prytime Medical®, Boerne, TX, USA) were used and advanced 45-48cm into the vessel; balloon was then inflated with 8ml of water. Placement confirmation was achieved by direct visualization via opening of the thoracic cavity.

### Outcomes

Assessment of correct REBOA placement and any complications were recorded. Successful REBOA catheter placement was determined as balloon inflation in the descending aorta between the left subclavian artery and the celiac trunk (zone 1). Time spans to identification of the intended target vessel, successful puncture and sheath introduction, and balloon inflation were recorded. Timing was started after the lecture when handover of the materials to the participants was completed.

### Statistical methods

Data were presented as absolute number (n) and percentage (%), or median and interquartile range (IQR), as appropriate.

For main analyses, between-group differences in the outcomes of interest were assessed by Chi-Square test or Mann–Whitney-U test, as appropriate; *p*-values below 0.05 were considered statistically significant. Time to balloon inflation was censored at 600 s. (i.e., 10 min) in cases of unsuccessful placement attempts or incorrect catheter positioning.

For secondary analyses, time spans elapsed were compared between groups in cases of successful correct catheter placement only. For exploratory post-hoc analysis, differences in time consumed until vessel visualization and until sheath introduction were compared between successful and unsuccessful attempts in the group with lower success rates. Vessel visualization was defined by participants’ impression having identified the correct vessel.

If significant differences were found, receiver operation curve (ROC) analysis was used to identify possible cut-off values to predict unsuccessful attempts.

All analyses were performed using IBM SPSS Statistics 28.

## Results

A total of 80 study participants were recruited and allocated to 40 open cut-down approaches and 40 percutaneous ultra-sound guided approaches. Information on successful puncture and cannulation and documented time spans were available in all cases. Specifics of medical specialties and training status of the participants are given in Table1.

### Main analysis

Successful placement of the REBOA catheter was achieved in 18 out of 40 cases (45%) using a percutaneous ultrasound guided technique and 33 out of 40 times (83%) using the open cut-down approach (*p* < 0.001) (Table 2).

**Table 2:** Success rates of and times elapsed for placement attempts of REBOA catheters compared between the percutaneous ultrasound-guided technique group and the surgical cut-down access group.

	Percutaneous ultrasound-guided technique	Surgical cut-down access	*p*
*n* of procedures	40	40	
Successful placement of REBOA catheter (n, %)	18 (45%)	33 (83%)	< 0.001
Times to [seconds] (median, IQR)			
Vessel visualization	36 (18–73)	136 (97–175)	< 0.001
Vessel puncture	117 (56–213)	183 (156–219)	< 0.001
Balloon inflation	375 (240–600)	288 (244–379)	0.08

IQR—inter-quartile range, n—number, REBOA—resuscitative endovascular balloon occlusion of the aorta.

Median times compared between the percutaneous ultrasound-guided technique and the surgical cut-down approach were 36 (18–73) s versus 117(56–213)s for vessel visualization (*p* < 0.001), 136 s. (97–175) versus 183 s. (156–219) for vessel puncture (*p* < 0.001), and 375 (240–600)s versus 288 (244–379) s for balloon inflation (*p* = 0.08). (Table 2).

Time for actual REBOA catheter placement after vessel access, i.e., the timespan between vessel puncture and balloon inflation was not significantly different between the ultrasound-guided technique [121 (82–174) s] and the cut-down approach [90 (61–132) s] (*p* = 0.122).

### Secondary analyses

Median times in cases of correct REBOA catheter placement compared between the percutaneous ultrasound-guided technique and the surgical cut-down approach were 18 (15–33) s versus 131 (95–165) s for vessel visualization (*p* < 0.001), 58 (36–170) s versus 178 (157–211) s for vessel puncture (*p* < 0.001), and 226 (149–315) s versus 284 (233–359) s for balloon inflation (*p* = 0.03) (Table 3).

**Table 3:** Times consumed for ***successful*** placement of REBOA catheters compared between the percutaneous ultrasound-guided technique group and the surgical cut-down access group.

	Percutaneous ultrasound-guided technique	Surgical cut-down access	*p*
*n* of procedures	40	40	
Time to [seconds] (median, IQR)			
Vessel visualization	18 (15–33)	131 (95–165)	< 0.001
Vessel puncture	58 (36–170)	178 (157–211)	< 0.001
Balloon inflation	226 (149–315)	284 (233–359)	0.03

IQR—inter-quartile range, n—number, REBOA—resuscitative endovascular balloon occlusion of the aorta.

Successful REBOA catheter placement was significantly more likely in the absence of arteriosclerosis [39 out of 53 (74%) vs. 12 out of 27 (44%), *p* = 0.01], whereas absence of obesity was not associated with a significantly higher success rate [34 out of 49 (69%) vs. 17 out of 31 (55%), *p* = 0.187].

Reasons for unsuccessful placement of the REBOA catheter were impossibility of insertion due to peripheral artery disease [6 out of 29 (21%)], vessel perforation and misplacement during puncture [7 out of 29 (24%)], venous puncture and insertion into inferior vena cava [6 out of 29 (21%)], or not identifiable vessel [10 out of 29 (34%)].

### Exploratory analyses

Within the group of percutaneous ultrasound-guided attempts, median times for successful and unsuccessful attempts were 18 (15–33) s versus 53 (35–87) s (*p* < 0.001) for vessel visualization and 58 (36–170) s versus 155 (89–236) sec. for vessel puncture (p = 0.08), respectively. (Figure S1 and S2—Additional File 1).

ROC analyses yielded best results for time to vessel visualization at 23 s. (sensitivity 0.91, specificity 0.67, AUC 0.83) and 90 s. (sensitivity 0.77, specificity 0.61, AUC 0.74) for time to vessel puncture, respectively. (Figure S3—Additional File 1).

## Discussion

In this prospective observational case control study using a cadaveric training model, ultrasound-guided puncture and surgical cut-down of femoral vessels demonstrate significant differences in success rates and time spent for REBOA catheter placement.

Success rate for REBOA placement is nearly twice as high using a cut-down approach compared to the ultrasound-guided technique. This might lead to the conclusion that this technique was the superior approach. However, when comparing times consumed, the ultrasound-guided approach compared to the cut-down approach requires significantly less time until balloon inflation, allowing for faster bleeding control and potential of hemodynamic benefit.

REBOA can be a life-saving procedure in exsanguinating trauma patients^16^. Broad consensus now underlines its role in the management of trauma resuscitation^17^. However, despite more than 600 publications on the subject, high-quality evidence concerning indications, complications, and outcomes is still lacking. The potential benefit of REBOA in trauma patients can be limited by complications as well as the elapsed time for catheter placement^8,18^.

On a procedural level, the rate limiting step in placement has been shown to be timing and safety for femoral artery access^8^. To our knowledge, this is the first study conducted in order to elucidate profiles of different techniques in femoral vessel access for REBOA catheter placement.

Our results show that clear and prompt identification of the targeted femoral artery are crucial for safe and timely access to femoral vessels for REBOA placement, with both techniques proving different advantages. Ultrasound-guided placement of different lines for vascular access, both arterial and venous, are nowadays deemed standard of care in critical ill and emergency patients^12^. As patients with severe bleeding after trauma can deteriorate rapidly and placement of those lines gets more difficult over time, early access is crucial^8,19^.

Our study data, in line with other reports, show that if vessels can be identified on ultrasonography vascular access can be established within 2.5 min, but failure to identify the correct vessel was the main reason for complications in more than half of cases. Furthermore, our data show that attempts for clear vessel identification using ultrasonography of approximately half a minute or more are predictive of the inability to place the REBOA catheter correctly using ultra-sound guided technique.

The DIRECT-IABO investigators report time to access to be directly associated with survival for patients undergoing REBOA^20^. If first line placement of a 7 French vascular sheath or a smaller device for arterial pressure monitoring (allowing for the possibility of using Seldinger’s technique to introduce a larger sheath at a later stage) should be used, is yet to be determined.

We thus suggest that arterial access should be obtained early during treatment of patients with potential benefit of REBOA, in order to prevent harm from complications and unnecessary time-consuming attempts once patients collapse and safe sonographic vessel visualization becomes more unlikely. Crally et al. have come to the same conclusion in their study, by identifying the time to catheter placement as a crucial step for improving outcome for this procedure^21^.

If patients already present in extremis, rendering visualization via ultrasonography uncertain within a timely fashion, a cut down approach to the femoral vessels should be performed. This conclusion is in line with other reports stating that if cardiac arrest is present or imminent cut down may be safer for femoral access^22^. Notably, resuscitative thoracotomy and aortic cross-clamping remain possible options in these patients. However, no clear evidence is available to prefer one method over the other^4^. Furthermore, the conversion from open aortic cross-clamping to REBOA may be an option in certain circumstances if providers’ training and expertise allow^23^.

Biological age and premorbid status have to be taken into account when considering different approaches to femoral vessel access. Peripheral Artery Disease, prior surgery or treatment in the anatomical area of interest may be more prevalent in older individuals. The investigated cadavers in this study, with a median age of 76 years, may overrepresent this age group, potentially skewing results towards one approach. This may limit generalizability and applicability to certain patients, since on the one hand, trauma and especially exsanguination is the most imminent threat to life in adults younger than 49 years^24^. On the other hand, due to demographic changes, the age of severely injured patients is steadily increasing^25^ and thus the investigated cadavers and their specifics regarding age related comorbidities may represent emerging challenges in trauma care.

Because of the high failure rate of ultrasound-guided techniques for REBOA catheter placement demonstrated by our study as well as previous studies^8^, we suggest practitioners, regardless of medical specialty, should be trained in both techniques when centers aim to perform REBOA during treatment of exsanguinating trauma patients. This conclusion is in line with previous studies trying to elucidate which medical specialty should be performing REBOA catheter placement, which have been unable to produce clear recommendations, but emphasize proper training^26^.

## Limitations

This study was conducted in a cadaveric training model with its known downside of not reflecting conditions in a living human being reliably, especially the absence of pulsations and missing backflow. Since we only used freshly cooled cadavers it still seems reasonable to translate results to some extent into real-life, as patients in severe shock after major blood loss and patients with traumatic cardiac arrest are considered prime candidates for REBOA catheter placement. Another downside was that the cadavers presented with a high rate of arteriosclerosis, a condition not usually present in the young exsanguinating trauma patient. With a rising number of trauma amongst the elderly and demographic changes, these findings still need considering and further research should address this topic as well.

Missing arterial pulsation and vasculature tone might have been unrealistic conditions leading to difficulties in vessel identification, especially for the ultrasound-guided technique. Anyhow, in comparison to embalmed bodies, fresh cadavers offer a genuine tissue handling and a variety of anatomic and pathologic variations.

Another limitation of this study was the lack of cross-over design for different medical specialties performing different techniques. Basic familiarity with the equipment used could have been a bias both in timing and accuracy, rendering medical specialty training a remaining potential for bias in our analyses.

## Conclusion

Our study shows that access to the femoral vessels for REBOA catheter placement in a resuscitation scenario is safer when performed by cut-down and direct visualization but can be performed faster by an ultrasound-guided technique when vessels can be identified clearly and rapidly.

### Supplementary Information

Supplementary Information.

## Data Availability

The datasets used and analyzed are attached in table form to this submission.

## Abbreviations

AUC: Area under the curve
BMI: Body mass index
IQR: Interquartile range
REBOA: Resuscitative endovascular balloon occlusion of the aorta
ROC: Receiver operating characteristics
