# Supplementary material for: Percutaneous ultrasound-guided versus open cut-down access to femoral vessels for the placement of a REBOA catheter
Source: Sci Rep. 2024 Apr 20;14:9111. doi: 10.1038/s41598-024-59778-x (PMC11032382; doi:10.1038/s41598-024-59778-x)

ADDITIONAL FILE 1

Percutaneous ultrasound-guided versus open cut-down access to femoral vessels for the placement of a REBOA catheter – a cadaveric study

Peter GRECHENIG, MD [1*]

peter.grechenig@stud.medunigraz.at, https://orcid.org/0000-0002-8226-7803

Paul PUCHWEIN, MD [1*]

paul.puchwein@medunigraz.at, https://orcid.org/0000-0003-2302-1050

Nicolas Rene EIBINGER, MD [1]

nicolas.eibinger@medunigraz.at, https://orcid.org/0000-0002-8921-1538

Amir KOUTP, MD [1]

amir.koutp@medunigraz.at, https://orcid.org/0000-0001-5992-4708

Paul ZAJIC, MD PhD [2]

paul.zajic@medunigraz.at, https://orcid.org/0000-0003-3097-1994

Gerald HÖFLER, MD [3]

gerald.hoefler@medunigraz.at, https://orcid.org/0000-0002-9056-3063

Barbara HALLMANN, MD [2]

barbara.hallmann@medunigraz.at, https://orcid.org/0000-0003-0230-9039

[1] Department of Orthopaedics and Trauma Surgery, Medical University of Graz, Graz, Austria

[2] Department of Anaesthesiology and Intensive Care Medicine, Medical University Graz, Graz, Austria

[3] Diagnostic and Research Institute of Pathology, Medical University Graz, Graz, Austria

* These authors contributed equally to this study and manuscript

Corresponding Author

Barbara Hallmann, MD
Department of Anaesthesiology and Intensive Care Medicine
Medical University Graz
Auenbruggerplatz 5
8036 Graz, Austria.
Telephone: +43 664 8218752
E-Mail: barbara.hallmann@medunigraz.at

Table S 1 Characteristics of cadavers

Age [years]	nutritional state	height [cm]	body weight [kg]	BMI [kg/m²]	sex	arterio-sclerosis
85	cachectic	163	50	19	female	no
77	adipose	178	81,6	26	male	yes
60	normal	176	80,4	26	male	no
50	adipose	175	118	39	male	no
85	normal	168	72,8	26	female	yes
70	normal	180	93	29	male	no
54	adipose	160	96,8	38	female	no
78	adipose	155	79,6	33	female	yes
29	normal	177	84	27	male	no
55	normal	183	95	28	male	no
62	normal	165	70,2	26	female	yes
77	normal	180	78,4	24	male	no
83	adipose	167	99	35	male	yes
76	normal	167	75	27	male	yes
84	adipose	181	93	28	male	no
77	adipose	177	79	25	male	yes
62	normal	172	82,4	28	female	no
54	adipose	163	94	35	female	no
29	normal	179	85	27	male	no
83	normal	184	79	23	male	no
77	adipose	160	85	33	female	yes
65	normal	170	73	25	female	no
80	adipose	183	97,4	29	male	yes
80	adipose	183	97,4	29	male	yes
74	normal	169	70,1	25	female	no
79	normal	177	80	26	male	no
62	normal	170	80,4	28	male	no
82	adipose	175	87	28	female	yes
79	normal	180	77	24	male	no
80	adipose	171	93	32	male	no
77	normal	173	80	27	female	yes
85	normal	182	88	27	male	yes
79	normal	168	74	26	female	no
68	normal	180	75	23	male	yes
73	normal	177	80	26	female	no
63	normal	177	74,4	24	male	no
77	normal	178	84	27	female	no
81	adipose	178	91	29	male	yes
70	normal	180	93	29	female	no
54	adipose	160	96,8	38	male	no
62	adipose	165	70,2	26	male	yes
76	normal	167	75	27	female	no
70	normal	178	75	24	male	no

Figure S 1 Scatter plots of time to vessel visualization [seconds] (x-axis) and incorrect placement [yes or no] (y-axis).

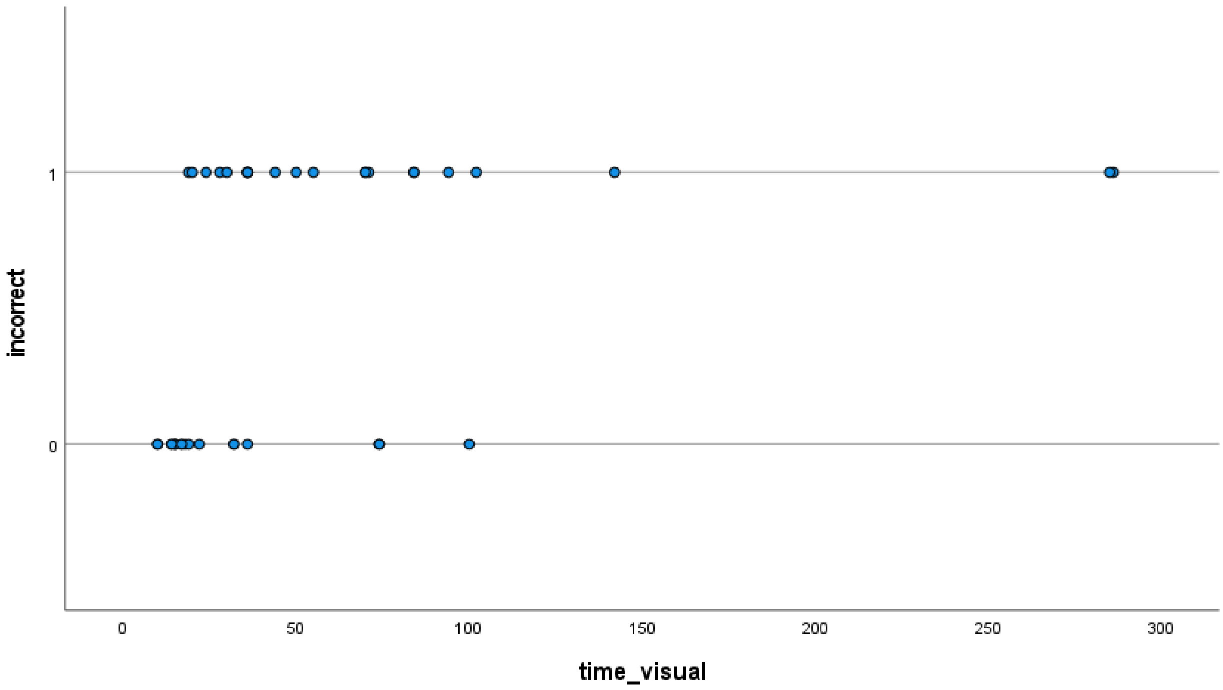

Figure S 2 Scatter plots of time to vessel puncture [seconds] (x-axis) and incorrect placement [yes or no] (y-axis).

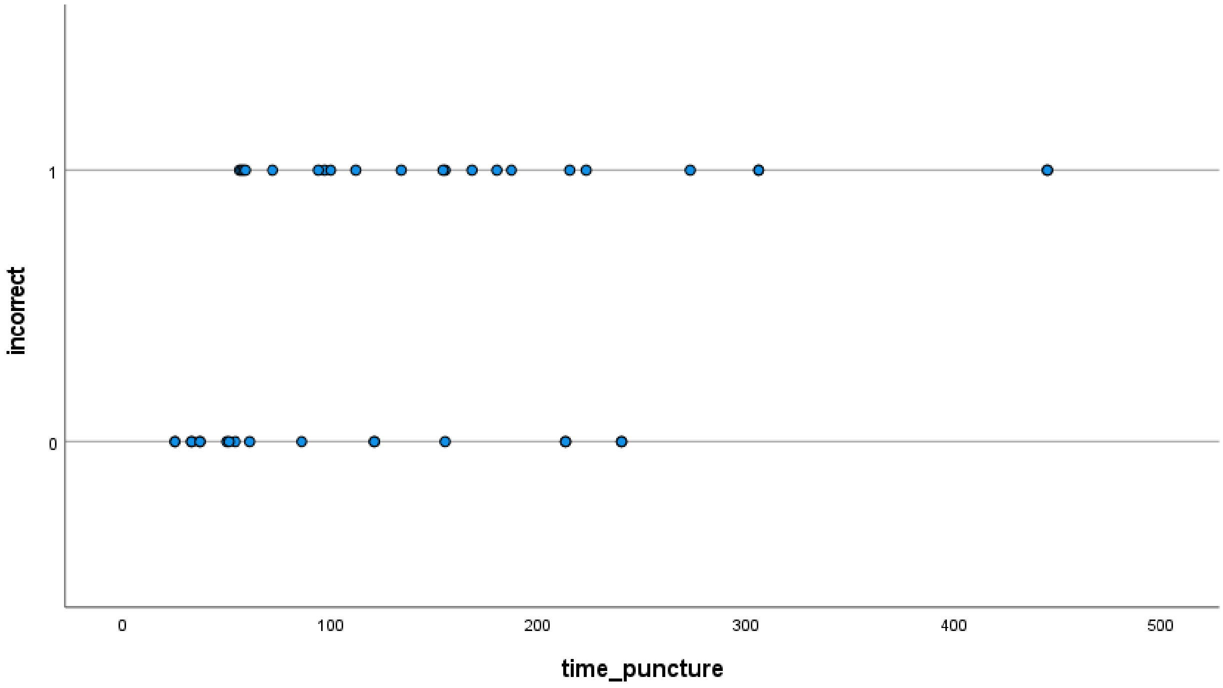

Figure S 3 Receiver Operating Characteristics curves for failed REBOA placement as state variable and time to vessel visualization or time to vessel puncture as test variables, respectively.

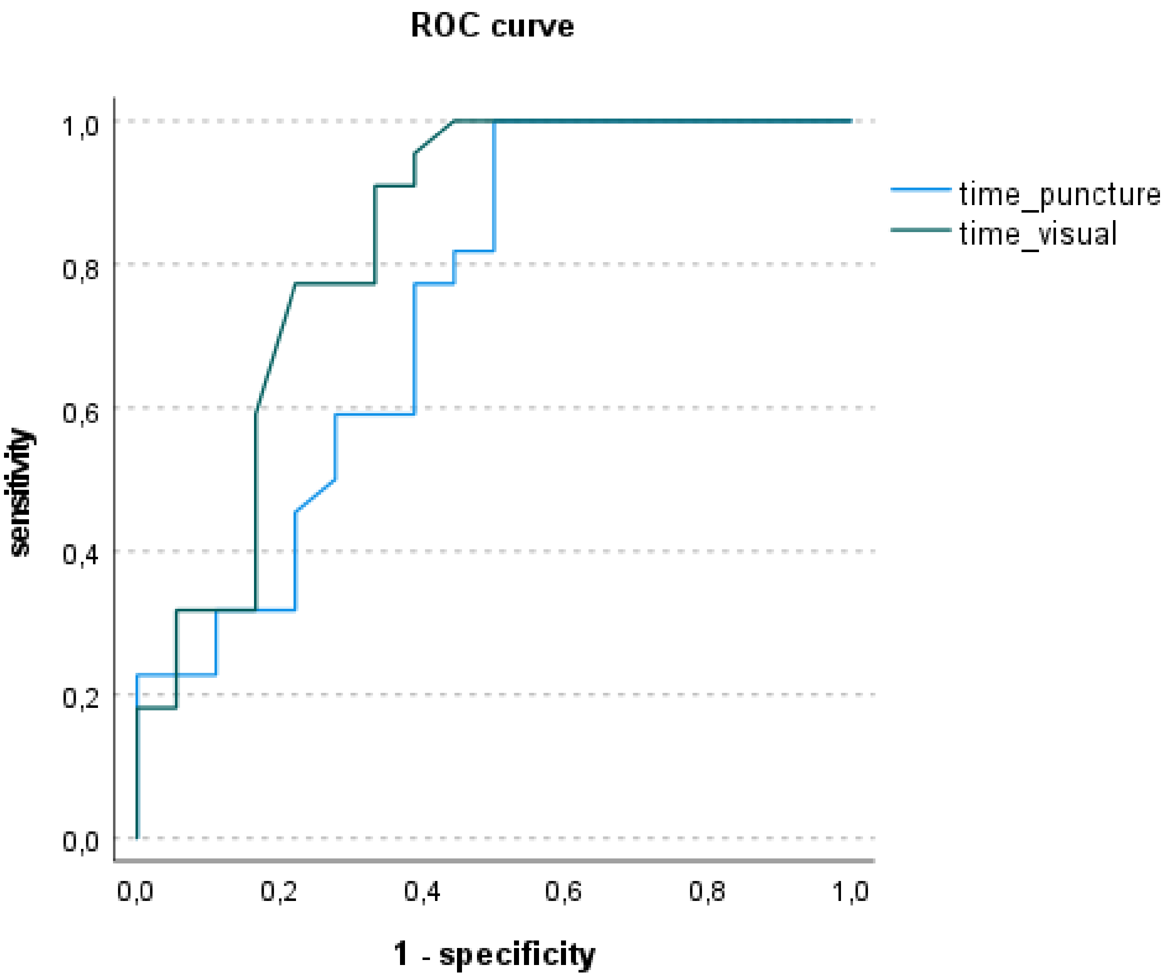

Supplement: Supplementary file 1 — Supplementary Information. [file 41598_2024_59778_MOESM1_ESM.pdf]
